# Thoughts on the Pathology and Treatment of Hydrocephalus

**Published:** 1827-07

**Authors:** N. Chapman

**Affiliations:** Professor of Medicine in the University of Pennsylvania.


### HYDROCEPHALUS.

*Thoughts on the Pathology and Treatment of Hydrocephalus.* By N. Chapman, M.D. Professor of Medicine in the University of Pennsylvania. (Condensed from the Philadelphia Journal.)

The earliest division of Hydrocephalus was into Externus and Internus, the water being supposed, in the latter form of the disease, to be situated between the cranium and its integuments, and in the former within the brain itself. But the distinction has long been abandoned. Cases of watery intumescence of the scalp do occasionally occur, which, however, are to be regarded as anasarcous affections, or serous depositions, in the cellular tissue of these parts. The ancients, though well acquainted with the first, seem not to have observed the second species, or at least they have left us no description of it. By Petit it was originally noticed, nearly a century ago, and not a great while afterwards by Whytt, of Edinburgh, who has delineated the case with such precision that later writers have been content to do little more than copy from him, with some amplification.

This is a disease chiefly incident to children, and, according to Morgagni, more to girls than boys; though the contrary is affirmed by Golis, a very authoritative writer on the subject. It is of rare occurrence in much more advanced life, though it is to be met with in persons beyond the meridian of existence; and I have seen several instances of it in females approaching the season of puberty, brought on by a translation of action from the uterus to the encephalon, by a vicarious assumption of the office of menstruation. There is reason, indeed, to presume that it is of more common existence than is supposed in adults, and especially among individuals liable to headaches, several such instances having come under my own observation.

Two causes have been assigned for the wider prevalence of the disease in children: the first is the disproportioned size of the head at this period, as well as a greater laxity or softness and vascularity of the brain, consequently producing an undue afflux of blood, with an increased disposition to effusion; and the second, the exposure at this age to falls, blows, and other accidents, operating as remote or exciting causes. It may be remarked, as resulting from this want of symmetry or just proportions, that children are *top heavy*, and hence, in all their tumbles, are very apt to strike the head. Yet, whatever share these circumstances may have in predisposing to or exciting the disease, I am inclined to believe that they are less than a certain condition of the chylopoietic viscera, which lays the foundation of nearly all our early morbid affections. Abernethy.

It is now well determined that the causes of hydrencephalus are such as act directly on the brain, or indirectly through the digestive organs; and hence it may be considered either as a primary or secondary affection.

To the causes already cited as appertaining to the first form of the disease, may be added tumors, schirrosities, ossifications, and various other derangements of cerebral structure; and to the second, the irritation from worms, or sordes of different kinds, habitual constipation, as well as whatever induces gastro-enteritic fever. It is by no means uncommon for our autumnal fevers, or the bowel affections, and cholera infantum particularly, thus to terminate; and it is scarcely less usual to meet with it as the sequel of catarrhal fever, when the mucous tissue of the primæ viæ is involved. Between that tissue and the arachnoid membrane there is a very close sympathy, and a state of irritation in the one is apt to be felt by the other, inducing acute pain in the head, then stupor, and finally effusions.

Moreover, it is sometimes occasioned by the sudden suppression or repulsion of acute and chronic eruptions, as the exanthematic, though particularly of crusta lactea, tinea capitis, and those discharges behind the ears common to children during dentition, and which may primarily disorder either the brain or the primæ viæ. There is no disease, in fine, which expends much of its force on the brain, that may not end in such effusions. Modern pathologists have, on this account, pretty generally ceased to consider it as an idiopathic or distinct affection, and have come to the conclusion that it is nearly always, if not uniformly, an effect merely of some pre-existing disease, having this ultimate tendency.

In some cases, the stage of predisposition, or irritation, is exceedingly insidious; so much so as to elude suspicion, where proper vigilance is not practised. The child will complain of languor, as if from fatigue; has a capricious appetite, thirst, torpid bowels, or loose unnatural stools, sometimes white and glutinous, with a mixture of green; high-coloured scanty urine; pale collapsed countenance, with a dark line under the eyes; furred tongue, harsh skin; some uneasiness of the head, which is more tenderness of the scalp than positive pain; tumid or rather puffy abdomen, with a sense of oppression about the pit of the stomach and seat of the duodenum, attended by pain on pressure. The child is fretful and peevish; the sleep is disturbed by startings, or general restlessness, and by day and by night is altogether uncomfortable. This state may last for an indefinite length of time, from one to several weeks; but, sooner or later, the phenomena of excitement, or phlogosis, begin more distinctly to be disclosed, in the shape of a febrile movement. It is now that we have nausea or vomiting, costive bowels; sometimes a dry florid tongue, though more generally moist and furred; hot skin, flushed face, acute headache, or pain and stiffness of the nape of the neck; or rheumatic affections of the joints, or soreness in the hands and feet; throbbing of the temporal arteries; strange sensations, or noises in the ears, as tinnitus aurium, the rushing of wind, or the falling of water, or the ringing of bells; much aversion to light and to sounds; dry nostrils, picking of the nose; and occasionally a pretty active, and sometimes irregular pulse. The fever is remittent, abating in the morning, with a sensible exacerbation at night.

Continuing in this way for a few days, we shall discover, with less general excitement, the approach of heaviness, attended by a knitting of the brows and a scowling expression of countenance. The pupil of one or both eyes is dilated or contracted; the pulse becomes slower, and sometimes intermits; the bowels unrelentingly constipated, or occasionally disordered, the stools being watery, or of a gelatinous consistence and clay-coloured, mixed with green scybala, the whole surface covered with an oily slime. The urine is still more deficient. Though for a time the sleep seems to be profound, it is still interrupted by frequent moanings, and startings, and grinding of the teeth. When aroused, there is at the moment partial delirium, manifested by a wild, distracted eye, incoherent mutterings, unusual behaviour, or quite irrational conduct. It often happens, at this or an earlier period, that a dry, teazing cough arises, seemingly from gastric irritation; or, when the case is associated with catarrhal fever, as formerly noticed, much mucus or phlegm, with sometimes wheezing and rattling, as in bronchitis.

This state of things advances on to the stage of oppression, owing probably to effusions. The pulse gradually becomes weaker, smaller, and more accelerated, till it is thready and can hardly be counted. There is squinting, more widely dilated or contracted pupils, rolling of the head, automatic or unmeaning tossing of the hands, low delirium, spasms or convulsions, usually of one arm or leg, or of the muscles of the face. No inconsiderable difficulty of deglutition also exists; and the respiration is frequent and laborious, with a lengthened pause between each expiration; and not unfrequently involuntary alvine and urinary discharges, the latter prodigiously copious, and mostly pellucid.

In this insensible condition the child will lie for days, exhibiting an afflicting spectacle. Emaciated considerably in some instances, its aspect is still more altered by the effects of its sufferings. The face is pale, the eyes are sunken, though half open with a film on the surface; the temples are hollow, the nose contracted, the forehead glazed, or moistened by a cold dewy perspiration. Death usually takes place in some convulsive struggle, in ten or fifteen days.

As I have described it, such is the tenor of the disease; but, like all others, it is infinitely diversified. Cases do occur, some of which I have seen, without any premonition, ending fatally in two or three days. The child, apparently well, is seized with fever, attended by gastric or cerebral distress, or both, soon becomes comatose, and dies convulsed. In other instances I have witnessed the same result from a sudden metastasis or retrocession of eruptions; and these anomalies have been so often presented, that they enter into some of the recent classifications of the disease, under the title of *water-stroke*, from the effusions in the brain detected after death.

Much more commonly, however, do we meet with it merely as the effect of protracted, low, abnormal, diminutive fevers, where little is to be anticipated from preceding symptoms. It as often, too, occurs in feeble and cachectic states of the system, with few or no precursory signs afforded by the pulse or topical excitement.

It were easy to multiply, to nearly any extent, descriptions of the various guises and aspects under which hydrencephalus is presented. But details of this sort are inconsistent with my design, which aims only at a very concise view of the subject.

Concerning the *diagnosis* in this disease, there is sometimes not much difficulty. Cases which most closely resemble it are some of the typhoid fevers, and those proceeding from worms, and torpid or depraved conditions of the bowels, or dyspeptic states of the stomach, where food, imperfectly digested, accumulates and lies oppressively. Yet it must be confessed that often even the most experienced practitioners are exceedingly perplexed, from those cases having so many symptoms in common by which they are very obscurely designated.

To arrive at a just conclusion, we must take a very minute survey of the disease, noting carefully its prominent appearances, in connexion with its previous history. The symptoms particularly to be attended to as chiefly distinctive, are the inclination to vomit, the constipation, or the aspect of the stools when procured,—the state of the urinary discharge,—the aversion to light, the noises in the ears, the dilatation or contraction of the pupils, the strabismus,—the slow irregular pulse,—the rolling of the head, the tossing of the hands,—the coma,—the impeded deglutition, and short interrupted respiration. There is another circumstance highly characteristic. Children can bear only the recumbent posture: taken up, they are immediately disposed to faint, or to be sick, and cry or scream, as if from extreme suffering.

Even, however, after this careful perquisition, and with all the lights derived from the elaborate and minute investigations of this point, contained in some of the recent treatises on the disease, every candid physician will acknowledge that any near approximation to certainty is unattainable. Nor do I know that it is so desirable as it is deemed. Whenever symptoms so similar exist, the parts must be in nearly a similar condition, and demand essentially a similarity of treatment.

In forming our *prognosis* as to the probable issue of hydrencephalus, we should be not a little influenced by our conviction of the manner of its production, and the state of the system with which it is associated. Excited by irritation of the chylopoietic viscera, it will prove more manageable than when originally seated in the brain; and where the case is acute and inflammatory, occurring in sound, robust children, than the reverse, or the slow and lingering, in habits feeble and disordered by the diathesis formerly mentioned,—which latter, indeed, are scarcely medicable. Not less incurable are those sudden attacks denominated *water-strokes*. As regards, however, even the most tractable forms of the disease, still more depends on the stage of the case. By a timely and vigorous course of treatment, it may be often cured antecedently to the effusion, though very seldom after this has taken place. Doubtless it is owing to their referring to those different stages of the disease, that we find such opposite reports among practitioners as to the degree of its curability. Thus MONRO avers his utter inability to cure it, and RUSH his distinguished success.

There are certain symptoms which I have observed, under all circumstances, to be of bad import. These are great disinclination to be raised, with an inability to sit up, from giddiness and confusion of head, and particularly when attended with tinnitus aurium, or other sounds, or by deafness. Dilatation or contraction of the pupil, or squinting, is unpropitious; and so is pain in the neck, much more so than in the head. Coma, convulsions, blindness, *or copious discharges of pellucid urine*, or watery stools, voluntary or otherwise, are usually mortal signs. CHEYNE thinks differently as to the urinary discharge; but I am sure he is wrong, such evacuations of urine always denoting a decay of cerebral energy. They occur in hysteria, from frights, and whenever, in short, this state of the brain and nerves is induced. The loss of cerebral energy in these latter cases being only temporary, no serious harm results from it. But in hydrencephalus it is very different: by compression or otherwise, the vital power of the brain is so greatly and permanently impaired, that the symptom to which I have alluded is uniformly one of the most fatal character.

The favourable signs are, of course, in some degree the reverse of these. Those, however, which most unequivocally denote recovery are the subsidence of vascular irritation and the cerebral affections, composure of the stomach, very bilious or more natural evacuations, healthy urine, or an approach to it, or heavy deposits in it, with soft perspirable skin, and, above all, defluxions from the nostrils. The last is often the earliest and most certain harbinger of an auspicious change, proclaiming the restoration of natural secretory action.

Much confusion has been introduced into the *pathology* of hydrencephalus, by confounding the pre-existent morbid condition with the effusion resulting from it. The first step in the attainment of clearer views is to dissolve this connexion, and to contemplate the disease in each of these presentations. It is now generally conceded that dropsy is merely the consequence of an altered state of the vessels of the part in which it takes place, either the serous or the cellular tissue, and that such alteration is usually associated with the phenomena of inflammation; all the remote causes of dropsy, however diversified, operating to the production of this effect. Conceding this, it is not less true that these tissues may pass through the several states of this process without such an event; and most commonly, indeed, the termination of it is otherwise. The pleura, the pericardium, the peritoneum, at least, when phlogosed, extravasate ordinarily coagulable lymph, and the cellular membrane the same; having a tendency, however, greater than the serous tissues to the secretion of pus. As inflammation thus varies in its terminations, it must be susceptible of modifications, and we are led to inquire into the circumstances which give to it that peculiarity inducing hydropic effusions. That it consists not in the intensity or feebleness of the action solely, seems to me to be sufficiently established. The question is one of great obscurity, and to attempt to solve it by alleging, as has been done, that the inflammation is of a specific nature, is only to repeat a barren, unmeaning phrase. Little more do we know concerning it than that, in proportion to the diffusiveness and the superficiality of the phlogosis in the membrane, is the disposition to serous eliminations. Generally this proposition undoubtedly holds true. Can it be denied, for instance, that when the cellular membrane is topically, though deeply affected, phlegmon arises, commencing with adhesive and ending in suppurative inflammation, or under the other circumstances that œdema is not as uniformly induced? Equally is the doctrine appropriate to the serous tissues, as is illustrated by the evidence of dissections, nearly always meeting with lymphatic exudations in partial or isolated patches of inflammation, and serous effusions where it is wide and slightly spread over the surface. More than to any of its kindred tissues does this latter remark apply to the arachnoid, which, under such circumstances, most abundantly effuses serum, even when the phlogosis scarcely exceeds an erythism. Delicate in the extreme in its fabric, it is also peculiarly averse to take on the adhesive or suppurative process, and hence it is that, while these are pretty constant occurrences in the peritoneum, pleura, &c. serous effusions are as uniformly the product of its inflammations. Does not this fact go far to explain the more frequent termination of the affections of head in dropsy, than those of either of the other cavities?

But is inflammation always the necessary antecedent to dropsy, without which it cannot take place? Before this question can be answered satisfactorily, we must understand what is meant by inflammation. Modern investigations have shown that, instead of an elementary state of action, it is a complicated process, beginning with irritation, followed by congestion, and ending in that condition to which the term phlogosis is usually applied. Whether irritation simply is productive of effusion, seems doubtful; but that it is consequent on congestion in some way, can hardly be disputed. Not to insist on the well-known experiments of LOWER, where effusion speedily followed the tying of the vena cava and the jugular vein, we have the less equivocal fact of the occurrence of dropsy by the interruption of the circulation, from the resistance of diseased viscera, as the liver, spleen, &c. These are proofs habitually appealed to in support of the hypothesis that alleges the connexion of dropsy with congestion or obstruction of the circulation, and which, at a glance, would warrant the conclusion. But a more careful examination exposes the error, and readily reconciles them to the doctrine of inflammation as the parent of the effusion. The cellular and serous tissues are those only in which such an effect ordinarily takes place, and, in such instances as cited above, it is demonstrable that the capillaries of the contiguous tissue becomes irritated into phlogosis.

But why does not the phlogosis of these tissues invariably lead to effusions? The question has, in part, been already answered; and, in further elucidation of it, I shall now add, as at least a probable conjecture, that it is not inflammation alone which induces genuine dropsy. Essentially associated with this condition, the vessels however assume a secretory power, by which a peculiar fluid is elaborated, very distinct in its properties from the serum, or any other of the constituents of the blood, and which faculty is sometimes continued long after the entire subsidence of that state whence it was derived. Did the effusion solely depend on the phlogosis, they should simultaneously cease; but it is very often otherwise, forming confessedly the principal difficulty in the cure of the disease. Certain cachectic states of the system favour the acquisition of this power by the vessels, so much so that we have emphatically an *hydropic diathesis*.

Thus far all seems to me very manifest. Cases of dropsy, and particularly of œdema, do however occur in conditions of such extreme debility, even of absolute exhaustion, that it is very difficult to embrace them within the same theory. It is certain that sometimes, though there is great general weakness, local excitement may prevail, and the effusion be the result of it: but on other occasions it happens where little or no grounds are observable for any such suspicion from symptoms, or by subsequent autopsic inspections. Taking this to be correct, I am inclined to believe that either an excited or enfeebled vascular action may be productive of the effect. To do away undue activity of the circulation, nature is disposed, as a salutary expedient, to cause an hemorrhage, or to resort to some sort of extravasation, and, under the circumstances stated formerly, to a serous effusion. But in states of exhaustion her powers are so impaired, that she is unable to resist the escape of these fluids, which leak, as it were, out of the exhalents. It is in this way that we have hemorrhages in the advanced stages of low fevers, and dropsical accumulations in other diseases of expended vital energy. As was at one time maintained, there would seem to be a very close analogy between hemorrhage and dropsy in many respects, each being active or passive, and that the old notions on this subject have been too hastily exploded.

As to the circumstances influencing cerebral effusion, I have nothing to add to the observations previously made. It may be collected from these that I view it as the result of diffused phlogosis in the arachnoid, or perhaps sometimes of the interstitial cellular membrane, or in both tissues. Excepting in the instance stated, the disease itself consists in this inflammation, and the effusion is only one of the proximate effects. It has indeed been held that the latter, so far from constituting the disease, is not the principal, or even accessory, cause of death in the case; operating, on the contrary, to the protraction of life, by imparting to the brain a degree of tone which otherwise it would lose from the ravages of disease. Certain it is that water will remain in the ventricles,—or at least we have every reason to suspect its existence, in some instances, for weeks, or months, or years, without any very serious detriment. But here there is a gradual effusion, and the brain accommodates itself to the distention. Directly the reverse of this, however, happens when suddenly induced; a train of phenomena arising, conspicuously indicative of cerebral oppression. Taking place more gradually, it may often be remarked that there is such a subsidence of the symptoms as to create an expectation of recovery. This is a most treacherous calm. The excited vessels are relieved by effusion, and the case assumes a mitigated aspect; but, after a short interval, the extraneous fluid, as in other instances of dropsy, operates as a re-exciting cause, and the disease returns with exasperated force, particularly as regards the signs of oppression. Yet it is not less true that hydrencephalus may be strongly characterised without any effusion. To this point the evidence is most conclusive. Numerous cases are recorded where effusion apparently existed, which, on examination, exhibited no such appearances.

But though I place the proximate cause of hydrencephalus in a morbid action of the brain, I am not the less persuaded, as already intimated, that, in a large proportion of cases, it commences in a disordered state of the stomach, &c. To this conclusion I am conducted by the well-known association between these parts, and by various considerations which may be deduced from the history of the disease: some of its causes, the great disorder in the digestive organs, the tenderness in the region of the stomach and liver, the obstinate constipation, the character of the biliary secretion, the peculiarity of the stools denoting vitiation, all of which sometimes exist for weeks antecedent to the hydrencephalic appearances; and, lastly, the phenomena on dissection, proving the existence of no slight derangement in several of the abdominal viscera, and occasionally none in the brain itself.

This brings me to the *treatment* of the disease, and which, in order to be appropriate, must be adapted to the three stages it presents in its more ordinary inflammatory or febrile character.

The predisposition to it, whether seated in the brain or abdominal viscera, may generally be removed by those remedies which are found most successful in the arrestation of the early movements of fever, consisting of evacuations of the alimentary canal, sometimes by an emetic, though principally by purgatives, and particularly the mercurial, aided by a state of rest and quietude, low abstemious diet, with a strict adherence to the antiphlogistic plan in every other respect. The diseased state of the abdominal viscera continuing, small alterative doses of calomel or the bluepill should be resorted to; and, where any painful uneasiness in the head or tenderness of the epigastrium arises, leeches to the affected part may be most advantageously applied.

But the case having advanced so far as the development of positive phlogosis, the practice is required to be more energetic and decisive. It might be presumed that venesection is the leading and most important measure: that it is useful, cannot be questioned, though the loss of blood in any considerable quantity is rarely well borne, and in some instances, even with no small activity of the circulation, a single bleeding is followed by collapse and exhaustion. It therefore behoves us to proceed cautiously with the lancet. The application of leeches usually answers better, and these may and ought to be repeatedly employed; substituting, when they cannot be had, cups, or the opening of the temporal arteries.

Not less important is purging, on the almost unremitting continuance of which much depends. To generalise too closely, or to be led away by analogy, in the practice of our art, is among the greatest of evils. That there is in this case some peculiarity of action which renders it more submissive to copious intestinal evacuations than to large detractions of blood, I am entirely persuaded. This is a truth which I wish to impress as the result of my own observations, sustained by the suffrage of much higher authority. Not many, indeed, whatever may be their speculative differences as to its pathology, now deny the superior efficacy of this process in the disease. It is called for as well to divert blood from the head, as to arouse the torpid condition of the bowels, to remove the foul accumulations which they contain, and rectify the morbid secretions of the liver. This organ is often most materially affected in the disease. In many instances, when a hydrencephalic state of the brain was suspected, I have seen the disordered stomach, the dilated pupil, the comatose tendency, and other alarming symptoms, removed by very free evacuations. Cheyne reports a remarkable case to the same effect, in which relief was immediately afforded by the bringing away "two chamber-potsful of the most extraordinary collection of feces."

Emetics have, perhaps, been too much neglected in the disease. An irritated or oppressed stomach I have seen to bring on symptoms very imitative, at least, of hydrencephalus, and which were promptly removed by puking. This is sufficiently intelligible. But the same sort of affection will occur, and of unquestionable gastric origin, though there may be nothing in the contents of the stomach to which it can be traced. Even under such circumstances emetics sometimes prove useful; probably in the same way that they remove some other cerebral and nervous affections, as tic douloureux, headache, apoplexy, &c. and which I believe they do, in the first place, on the principle of revulsion, the strong impression on the stomach attracting the blood to that point, and next on the reaction of the system, establishing a more just distribution of it. To their use I was first attracted by observing the disposition in the disease alternately to affect the stomach and brain, and when the former was much disordered the latter became relieved. In hydrencephalic and other dropsical tendencies, they probably go further in their salutary influences, by changing the state of the secerning vessels which leads to effusion. Between hemorrhage and dropsy, I formerly mentioned, there is an analogy; and of the decided efficacy of emetics in obviating and suppressing the flow of blood, little doubt can longer remain.

Evacuations having been thus premised, blisters prove very serviceable. The application should first be made to the nape of the neck, and subsequently to the cranium, of a sufficient size to embrace the whole of it from the ears upwards, to remain on for twenty-four or thirty-six hours, or till suppuration of the scalp is induced, without which it is comparatively useless. It will be well, where the delay is admissible, to shave the head for some time previously to the application, so as to guard against strangury, which is very apt to occur from blisters in this situation, and may be prevented in the way I have stated. There is another advantage in early removing the hair, that we command a larger and better surface for the cold applications, and perhaps, by the loss of this warm covering of the head, directly mitigate the force of the disease. In many of the cerebral affections, acute and chronic, as certain fevers, cephalalgia, &c. it is perfectly ascertained that it is productive of prompt and unequivocal relief, and there is no reason why it should not prove useful in the case before us. I will only add that, as a general rule, it is better practice to anticipate the blister, by cold applications to the head, and stimulating pediluvia frequently repeated, or cataplasms to the feet and ancles of the same character.

This is, I believe, the best treatment in the early stages of the disease; and as to the final one, little aid can be rendered. Effusions in most instances have taken place, and, though in other cavities these are often taken up, experience teaches that here it seldom happens. No one has clearly demonstrated lymphatics in any portion of the brain, and by many their existence is denied: but surely these not having been satisfactorily ascertained is owing to the imperfection of our researches, as the phenomena of growth, not to mention other facts, sufficiently attest that they must belong to every organ and part of the animal machine.*

An absorbent is as necessary an ingredient in the composition of a living body as a blood-vessel; each being indispensably necessary to the execution of its primary and most important vital functions. Even, admitting, however, the

* Since the absorbing power of the veins has been so satisfactorily shown, the existence of lymphatics in the brain seems no longer necessary to explain the phenomena.—E.

existence of lymphatics in the brain, which I say cannot be denied, it is still not less true that they act very feebly and incompetently in the hydrencephalic affections; yet on this account we should not be discouraged from making the most strenuous efforts.

No plan of treatment, perhaps, holds out such prospects of success as the free employment of mercury. Even where effusion has not taken place it is serviceable, by changing the action of the vessels, provided it be properly reduced, and diverting the complaint from the head: but, if it exists, it is the only remedy entitled to the slightest confidence. This practice, commenced in England by DOBSON, in the year 1775, was soon generally adopted. There are not a few cases reported of its success: yet it must be confessed that for some time it has been losing reputation, and is now comparatively little employed, which I am inclined to suspect is owing to the timid and incompetent course adopted.

To be effectual, mercury must be applied in a very resolute manner: it should be exhibited in as large a quantity as the stomach and bowels will bear; and externally applied, in the shape of frictions, with the strongest ointment, most diligently and copiously.

Though often disappointed in my expectations from it, I have seen it of manifest service, and especially in two instances, the outline of which I shall give.

In 1814, I attended, in consultation with the late Dr. KUHN, a child of six years of age, who, having passed through the early stages of well-marked hydrencephalus, presented the phenomena of effusion. Conformably to his established practice, he resolved, if possible, to attain the specific influence of mercury, and with this view directed calomel to be freely exhibited, while the whole surface of the body should be rubbed twice a-day with the mercurial ointment, of double the strength of that of the dispensatories. Not content with this, he had gloves reaching to the armpits, stockings up to the groins, a belt around the abdomen, and a cap to the head, smeared with the unguent, kept constantly on; all which was perseveringly done till the fourteenth morning, when, on the appearance of a slight ulceration of the gums, a manifest improvement taking place, it was discontinued, after a consumption of fourteen pounds and a half of the ointment. Convalescence henceforward rapidly advanced, without any further inconvenience either from the remedy or the disease.

Not long afterwards, I saw occasionally with this distinguished practitioner, and the late Professor WISTAR, a child nearly of the same age under similar circumstances, in whose case this course of treatment was pursued, and with an equally happy result. Both patients are now living, and in full health.

It can hardly be supposed, after the admissions I have made, that these cases are cited to inspire any sanguine hopes in the success of the practice: but it will sometimes answer; and, under circumstances so desperate, what else can be done? Diuretics, so useful in other dropsies, are here utterly nugatory, and these are the only means which would seem to hold out any advantage.

Of the inflammatory form of the disease, I have said all which I deem necessary; but, in place of it, cases are not unfrequent, in a very opposite state of the system, such as I formerly described. Effusions, in many instances, take place before our attention is attracted to the cerebral affection, and we are aware how deplorable is such a state of things. Depletion by venesection is hardly ever admissible, though we may, in the early stage, now and then resort to leeches as palliatives of action; and a blister to the head should be applied. The chief reliance, however, is to be placed on moderate purging in the beginning, and afterwards in mercury alone, or possibly, as has been recommended, in combination with the squill or digitalis. But I have never seen a cure effected.

Most of these cases properly belong to the second division of the disease, or chronic hydrencephalus. The effusion being slow, so as to allow the brain to adapt itself to the deposition, I have known them to run on, with alternate remissions and exacerbations, for a great length of time. But the most decided examples of this species are congenital, or appear soon after birth, while the cranial bones are imperfectly united.

The brain here gradually yields to the distention from the accumulated fluid, till finally it loses its original shape, and apparently its organisation, becoming a mere sac enclosing the fluid. What is very extraordinary, in this condition the possession of the senses, as well as of the intellectual faculties, seems in a great measure to be retained. To the researches of Gall we owe the solution of this problem, who has shown that the brain, somewhat like the peritoneum, is a bag, deriving its compactness and solidity from its being folded up compressed, as it were, in the case which encloses it. By careful dissection, he has drawn out all these folds and involutions, and made the demonstration I have stated. The disease accomplishes the same thing, and, as there is no loss of substance in the brain, its functions in this state may be performed.

In the management of this species of the disease, medicine is so destitute of resources, that it must be resigned to surgery. Thirty-five years ago, Professor PHYSICK was nearly effecting a cure of one of these cases, by puncturing the membranes and drawing off the water. The same operation has since been performed by Dr. GLOVER, of Charleston; and in each instance a cure would seem to have followed, had there not been an interposition of accidental and adventitious causes to frustrate it.*

The history of these cases naturally leads to the inquiry, how far it might be proper to extend this operation to acute hydrencephalus? It, of course, should be reserved only for such instances as where an accumulation of water is clearly manifested, and the usual remedies had failed. I do confess that, under such circumstances, I should think it admissible; and I am not sure that it might not prove beneficial, or even a cure. As mentioned before, the blood-vessels being relieved by effusion, there is often, till it excites a reaction, a remission in the disease: it is at this period that an attempt should be made.

* For a case in which this operation proved successful, see Med.-Chirurgical Transactions, vol. ix.

